# High genetic diversity, clonal activation of hypnozoites and relapse of Plasmodium vivax isolates in low-transmission setting of Ethiopia

**DOI:** 10.21203/rs.3.rs-9080494/v1

**Published:** 2026-03-26

**Authors:** Hallelujah Getachew, Daibin Zhong, Kassahun Habtamu, Ashenafi Abossie, Assalif Demissew, Arega Tsegaye, Teshome Degefa, Chloe Wang, Guofa Zhou, Ming-Chieh Lee, James W. Kazura, Christopher L. King, Delenasaw Yewhalaw, Guiyun Yan

**Affiliations:** Jimma University; University of California, Irvine; Menelik II Medical & Health Science College; Arba Minch University; Ambo University; Jimma University; Jimma University; University of California, Irvine; University of California, Irvine; University of California, Irvine; Case Western Reserve University; Case Western Reserve University; Jimma University; University of California, Irvine

**Keywords:** Plasmodium vivax, amplicon deep sequencing, genetic diversity, multiplicity of infection, hypnozoite, relapse, pvmsp1 gene, Ethiopia

## Abstract

Recurrent infection is more common in *Plasmodium vivax* malaria. The recurrence of *P. vivax* can be due to recrudescence, reinfection, or relapse. To characterize the genetic signature of *P. vivax* genetic diversity and multiplicity of infection (MOI) were assessed using merozoite surface protein 1 gene (*pvmsp1*). A total of 370 blood samples were collected from 215 individuals visiting health facilities within Arjo-Didessa sugarcane plantations and it’s surrounding of Oromia, southwestern Ethiopia. All samples were subjected to amplicon deep sequencing of the *pvmsp1* gene. High population genetic diversity was observed—generating 67 unique haplotypes, haplotype diversity (Hd = 0.799), nucleotide diversity (ℼ = 0.044), and expected heterozygosity (HE) = 0.826. However, low MOI = 1.4 and 34.6% polyclonal infections. Of the 215 participant, 82 patients experienced one to five recurrent infections. In paired analysis of primary and recurrent episodes, high genetic homology (81.3%) was observed, with 55.6% of the homologous pairs sharing identical alleles. The high genetic diversity at population and low diversity at individual level likely driven by migrant workers introducing diverse parasite genotypes into a low-transmission setting. Most of the recurrent infections were relapses, as evidenced by shared alleles. The finding highlights the need for strengthening malaria surveillance and tailored intervention particularly for mobile population.

## Introduction

Unlike *Plasmodium falciparum*, which is largely restricted to Africa, *Plasmodium vivax* malaria has wide distribution and spatial heterogeneity at the global and local scales, with significant presence in Asia, Latin America, and in some parts of the Horn of Africa ^1,2^. The global cases of *P. vivax* declined from 10.6 million in 2013 to 4.2 million in 2020, but it resurged to 9.2 million in 2023 ^3^. In 2022, approximately 34.5% of global vivax malaria cases were attributed to Ethiopia, which accounted for 14% of the total malaria cases ^4^. In Ethiopia, *P. falciparum* and *P. vivax* parasites are co-endemic. The proportion of *P. falciparum* and *P. vivax* parasites reaches up to 60% and 40%, respectively ^5^. However, the percentage might fluctuate on the spatial and temporal scale ^6–9^.

*Plasmodium vivax* has been less responsive to malaria control and elimination efforts as compared to *P. falciparum* ^10^. Different factors contribute to this challenge; these include asymptomatic and submicroscopic nature of the infection, which causes a hidden reservoir for *P. vivax* transmission ^11^. On the other hand, mature *P. vivax* gametocytes appear in the bloodstream before the onset of clinical symptoms; therefore, silent infections may play a significant role in the onward transmission before patients seek treatment ^12–15^. Moreover, possessing latent liver stage (hypnozoite) causes reactivation of *P. vivax* infection occur weeks to months or years later of the primary attack. The recurrence of *P. vivax* can be due to treatment failure (recrudescence), new infection (reinfection), or relapse (reactivation of hypnozoites) ^16^. Genetic characterization of pre-treatment and post-treatment isolates by molecular genotyping methods such as PCR-based genotyping or next-generation sequencing has been used to distinguish between treatment failure, new infection, or relapse ^17–21^. Paired pre-treatment and post-treatment isolates can be classified as genetically related (homologous) or genetically distinct (heterologous) recurrence ^22–25^. Relapses may originate from reactivation of parasite clones homologous to the primary attack ^18,21,26,27^ or heterologous, making the task of identifying the relapse source difficult. Since homologous recurrence could be a treatment failure and heterologous recurrence could be a new infection. Studies of relapse have been hampered by reinfection due to the frequent finding of heterologous parasites at relapse, even in the setting of known relapse ^19,22,24,25,28^. This fundamental limitation currently impedes the accurate evaluation of anti-relapse interventions in clinical studies.

Molecular genotyping methods are used to study genetic diversity and complexity of infection (multiplicity of infection) of recurrent infections ^29^. However, the PCR-based genotyping methods have limitations on detecting the complexity of infection due to their lack of both sensitivity and specificity as compared to next generation sequencing, such as amplicon deep sequencing, which has the potential to overcome some of the shortcomings ^29^. High multiplicity of infection (MOI) is common in high transmission settings ^30^ due to two distinct phenomena, such as co-transmission and superinfection ^23^. Co-transmission occurs when an individual is bitten by a single mosquito that carries a polyclonal parasite genotype, while superinfections occur when an individual is bitten by two or more mosquitoes that carry unique parasite genotypes, and these unique parasite clones recombine in the mosquito to form multiclonal sporozoite, then multiclonal hypnozite and parasite in the bloodstream ^23^.

The evaluation of the evolutionary dynamics and genetic mechanisms of *P. vivax* malaria relies on the molecular markers with high diversity to estimate MOI accurately. Among these polymorphic antigens, *P. vivax* merozoite surface protein 1 (PvMSP1) is one of the most extensively studied markers ^27,31–36^. This protein, encoded by the *pvmsp1* gene, is critical for the parasite’s invasion of erythrocytes. This gene composed of nine variable regions that are separated by conserved blocks ^33^. The variable block 18, located within the 42 kDa region of *pvmsp1*, has been identified as the most polymorphic antigen ^37^. Consequently, this specific marker is particularly suitable for detecting and differentiating recurrent infections caused by distinct *P. vivax* strains. Therefore, this study assessed the genetic diversity and multiplicity of infection of *P. vivax* recurrence by the *pvmsp1* gene amplicon deep sequencing in the low-transmission settings of southwest, Ethiopia.

## Results

A total of 384 PCR reactions (370 samples and 13 samples were randomly selected and run-in duplicate). The 370 samples collected from 215 *P. vivax* positive patients, with a total of 8,250,523 joined reads were obtained by the fast-join program, of which 2,691,772 (32.6%) were successfully clustered by AmpSeqR with an average of 7,275 reads per sample at within-host cluster frequency > 2.0%. The *pvmsp1* amplicon generated an identical 309 bp fragment with 67 unique haplotypes. Among them, 26 *P. vivax* predominant haplotypes (the clone had the highest frequency within infection) were identified (see Supplementary Fig. S1). A nucleotide BLAST search was performed at the National Center for Biotechnology Information (NCBI), and 11 of the 26 unique haplotypes had a perfect match to GenBank sequences and > 99% sequence similarity for the others against distinct sequence from GenBank (see Supplementary Additional file 1 and 2)

### Haplotype diversity and population frequency distribution

**Figure 1:**
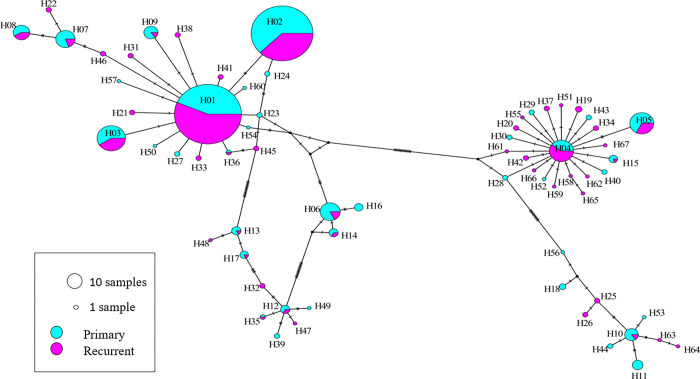
Median joining networks of *pvmsp1* haplotypes showing all variants detected (H01–H67). Frequency of haplotypes is indicated by circle size; circle fill color indicates primary (sky blue) and recurrent episodes (purple); numbers in brackets near the connection lines indicate the number of mutations

**Figure 2:**
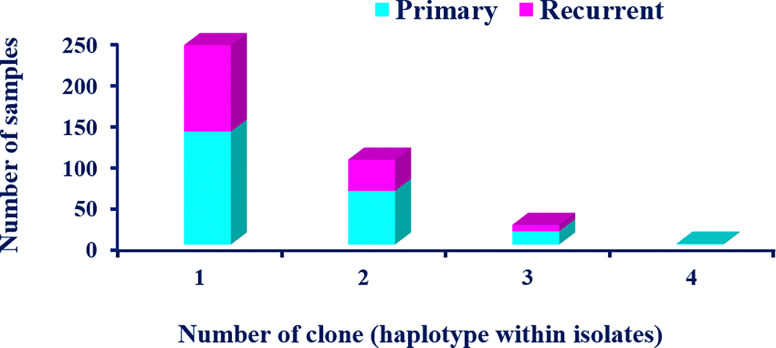
Multiplicity of infection in *P. vivax* among primary (sky blue) and recurrent episodes (purple)

**Figure 3:**
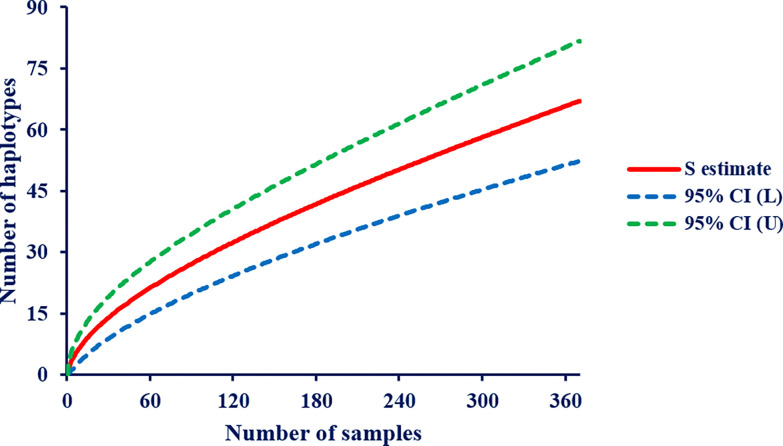
Sample‐based rarefaction curves for haplotype richness of *P. vivax*. The graphs show the rarefaction curve (S estimate, in red solid line) with 95% confidence intervals (CI, in dashed lines). L lower limit, U upper limit

The 67 unique *pvmsp1* haplotypes exhibited 57 variable (polymorphic) sites, including 3 singleton variable sites. The average haplotype diversity (Hd) and nucleotide diversity (ℼ) were 0.799 and 0.044, respectively. All 67 haplotypes successfully translated into complete amino acid sequences, yielding 52 distinct amino acid haplotypes. Eight (8) nucleotide haplotypes each appeared in at least 10 samples (see Supplementary Fig. S2), while 68.6% (46/67) of haplotypes appeared in only one individual sample, with within-host frequency ranging from 0.68 to 100%. Half of 55.2% (37/67) of the identified haplotypes were detected as minority variants (within-host frequency < 20%). Some of these minority variants were detected across multiple samples: H04, H05, and H14. Other minority variants detected formed part of the mutational path between the more common variants, as depicted in a median joining network based on sequence relatedness, adding support that they are true haplotypes and not a result of PCR or sequencing error (Fig. 1). Haplotype H02 was common in primary infection, and H01 was common in recurrent episodes. In paired primary infection and recurrent episodes, H01 significantly increased in recurrent episodes while H02 haplotype significantly decreased, H01 = 26.8% vs H02 = 30.1% and H01 = 42.6% vs H02 = 21.5%, *p* = 0.014 in primary infection and recurrent episodes, respectively. However, these two haplotypes were persistent throughout the 3 years follow-up duration. Polyclonal infections were detected in 34.6% (128/370) of the overall samples, ranging from two to four clones per sample, 39.0% (32/82) vs 29.7% (46/155), *p* = 0.145 in paired primary and recurrent episodes, respectively (Fig. 2). The overall Nei’s unbiased expected heterozygosity at this locus was HE = 0.826, representing an average 82.6% probability for two parasite clones harboring different *pvmsp1* haplotypes in the population; in paired samples of primary and recurrent episodes were 0.829 and 0.766, respectively. Estimates of allelic richness in all the 370 samples indicated that there was no clear plateau in accumulation curves (Fig. 3), suggesting more haplotypes would be expected to occur from increased samples.

#### Determination of relapse in P. vivax infection

**Figure 4:**
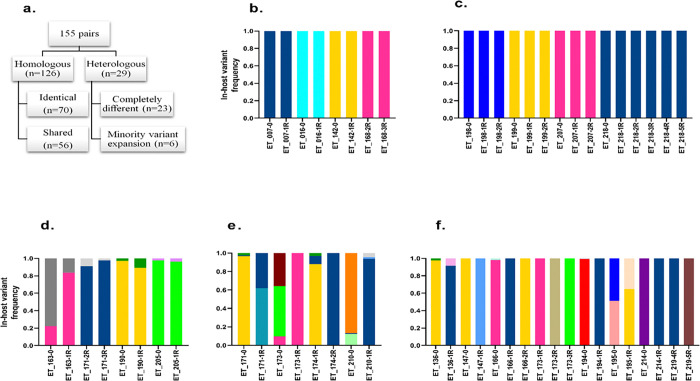
Homologous and heterologous pairs of 82 patients based on the overlap of *pvmsp1* variants in the recurrent and preceding infection. Unique haplotypes (alleles) are represented by specific colors across all samples. **a.** A total of 155 paired recurrent infections based on overlap of *pvmsp1* variants in the recurrent and preceding infection, **b.** Homologous pairs with clonal relapse in a single relapse episode, **c.** Homologous pairs with clonal relapses in multiple relapse episodes, **d.** Homologous identical pairs with two clones, **e.** Pairs exhibiting minority variant expansion (allele existing at <20% in-host frequency in the primary infection reappeared as the dominant or increased >20 in-host frequency at recurrence). **f.** Heterologous pairs with no overlap alleles at all. The x-axis indicate patient ID with 0 = primary infection, 1R = first recurrence, 2R = second recurrence, 3R = third recurrence and so on.

Of 215 study participants, 82 patients had one to five recurrent episodes were analyzed for relapse. A total of 237 samples from 82 patients (82 primary and 155 recurrent episodes samples) with 42 patients had one recurrence, 18 had two recurrences, 15 had three recurrences, 3 had four recurrences, and 4 subjects had five recurrences were analyzed. Among the 155 recurrent episode samples, 81.3% (126/155) showed homologous recurrence by amplicon deep sequencing, exhibiting the same or shared *pvmsp1* haplotypes, while 55.6% (70/126) of paired homologous recurrent samples shared identical alleles from the preceding episode (Fig. 4a) (see Supplementary Fig. S3). In the homologous pairs, 33 were single relapse episodes and 37 were two or more relapse episodes (Fig. 4b). From the single relapse episodes, six of them shared two identical alleles in both primary and recurrent episodes (Fig. 4d). In age-separated analysis, the relapse episodes in children under the age of five showed 12 out of 12 homologies to the preceding episode when the relapse episode occurred between 29 to 60 days after the primary infection, and it was significantly associated (*p* = 0.041). While 9 (81.8%) in children between 5–15 years of age and 12 (82.4%) in adults above 15 years of age showed homology in the same time frame (29–60 days) with no statistically different (Table 1). In addition, from 34 recurrent episodes between day 29 and 42, 61.7% (21/34) of CQ treated and 23.5% (8/34) of CQ + PQ treated group showed an homologous alleles to the primary infection with no significant difference (*p* = 0.574). While from 121 recurrent episodes above day 42, 35 of CQ treated and 23 of CQ + PQ treated group showed an identical alleles with no significance difference (*p* = 0.115). From 155 recurrent episodes, the remaining 29-paires samples were heterologous (Fig. 4a). The majority of the heterologous pairs (23/29) were completely different *pvmsp1* variants at recurrence (Fig. 4f), and 20.7% (6/29) heterologous pairs showed a pattern of minority variant expansion (the minor allele with in-host frequency < 20% in primary attack becomes dominant allele in the subsequent recurrent episodes) (Fig. 4e). In paired primary and recurrent episodes, 28 and 39 haplotypes (alleles) were observed, respectively (Table 2). Of these, 15 of the primary and 23 of the recurrent alleles were minor haplotypes existing at < 20% in-host frequency.

#### Minority Variant Expansion

**Figure 5:**
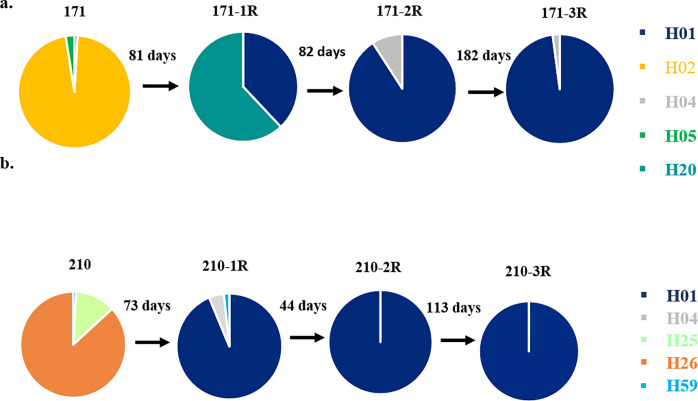
Minority variant expansion of *pvmsp1*haplotypes in patient 117 (**a**) and 210 (**b**) through four consecutive *P. vivax* recurrent episodes. Specific colors represent different haplotypes (alleles) and the number with days represent the time interval, which the next recurrence occurred. In both patient H01 haplotype becomes dominant in subsequent relapse.

In 20.7% (6/29) of the heterologous recurrences, the recurrence genotype displayed a pattern of minority variant expansion, in which a variant existing at < 20% in-host frequency in the primary infection reappeared as the dominant variant at recurrence (Fig. 4e). This type of pattern was illustrated in patient 171, the first and second recurrences occurred between 81–82 days interval; however, the third recurrence occurred between 182 days interval from the second recurrence. In this patient, the H01 haplotype increased its fraction through time and became the dominant haplotype (Fig. 5a). In the same way, in patient 210, the H01 haplotype increased through recurrence with different recurrent time intervals, such as day 73, 44, and 113 (Fig. 5b).

### Classification of recurrences as relapses

To estimate whether the recurrent pairs with shared allelic variants were relapse or not, a previously published method by Lin *et al.*, was used and the probability that the allelic variants would recur in the same person by chance or not was calculated, by taking the overall population prevalence ^19^. This probability of a recurrent genotype represents a new mosquito-inoculated infection or not. Thus, for the recurrent patient with x allelic variants and sharing a single allelic variant of prevalence y, the binomial probability ^38^ that this allelic variant is found by chance or not in a recurrent infection is calculated as follow 1−(1 − y)^x^.

A cutoff of 10% is used to classify recurrences as probable relapses due to from the reactivation of hypnozoites within the patient; if the probability of reinfection by the shared allelic variant(s) is ≤ 0.10, the recurrence is classified as a relapse, and otherwise, “indeterminate.” An overall relapse was 34.2% (53/155); however, from 126 homologous and related pairs, 35.4% (45/126), and from the 28 heterologous pairs, 27.6% (8/29) showed a probability of reinfection by the shared allelic variant(s) is ≤ 0.10. Among the 126 homologous and related pairs, 61.1% (77/126) and 17.2% (5/29) of heterologous pairs had reinfection probabilities of > 10%, placing them in the indeterminate category.

#### MOI of pvmsp1

**Figure 6:**
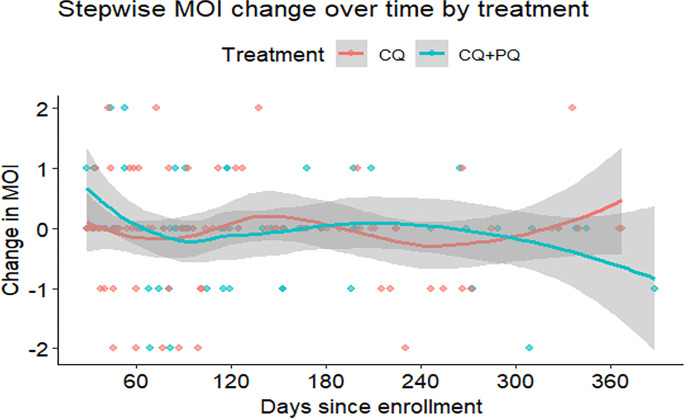
Change in MOI between CQ and CQ+PQ treatment group by time in paired recurrent and preceding *P. vivax* infection.

MOI was analyzed for 370 samples. The overall MOI in this study was 1.4 with 34.6% (128/370) polyclonal infection. In the paired primary and recurrent episodes sample of 237, 39.0% (32/82) of the infections were polyclonal in primary infection, with an average of 1.5 co-circulating variants, while 29.7% (46/155) were polyclonal in recurrent episode with an average of 1.35 co-circulating variants (Table 2). The median MOI in primary and recurrent episodes was 1, IQR: [1–2], *p* = 0.1044. The mean MOI of the primary infection was slightly higher than recurrent; however, no significant difference. The percentage of polyclonal infection was 27.3% (32/117) for asymptomatic and 38.3% (46/120) for symptomatic episodes, respectively. The average MOI in symptomatic episodes was 1.47 and 1.33 in asymptomatic, with the median MOI 1, IQR: [1–2], *p* = 0.077. In the same way, the polyclonal infection was 32.8% (44/134) in CQ-treated group and 33.0% (34/103) in the CQ + PQ-treated group, respectively. The same average MOI (1.4) in the CQ and CQ + PQ treated group, with the median MOI 1, IQR: [1–2], *p* = 0.936 detected. However, the change in MOI over time in CQ + PQ showed a decrease in MOI and a fluctuation in MOI in the CQ treatment group (Fig. 6). The polyclonal infection between male and female was also (36.2% vs 25.7%) with an average MOI (1.46 vs 1.27) and equal median MOI 1, IQR: [1–2], *p* = 0.062, respectively. The polyclonal infection between different age groups were 36.2%, 29.7% and 33.6% in children < 5, 5–15, and adults above 15 years of age. Similar average MOI (1.4) seen in the three age groups with equal median MOI value (MOI 1, IQR: [1–2], *p* = 0.793). The polyclonal infection, mean MOI, and median MOI between dry and wet seasons of *P. vivax* infection were 36.6% 30.6%, 1.4, 1, IQR: [1–2], *p* = 0.428, respectively.

In this study, the analysis of molecular variance (AMOVA) of *P. vivax* infection using the *pvmsp1* amplicon deep sequencing showed similar within-individual and among-individual genetic variation (50.2% vs 49.8%) in the overall samples. However, between primary and recurrent episodes, there was a difference within individual and among individual genetic variation. In primary infection, there was 58% within individual and 42% among individual genetic variation, while in recurrent episodes, there was 40% within individual and 60% among individual genetic variation (Table 3).

## Discussion

In this study, the amplicon deep sequencing of the *pvmsp1* gene provided a high-resolution view of the *P. vivax* genetic diversity and the biology of relapse in the low transmission setting of Ethiopia. Genetic diversity coupled with detailed paired-sample analysis revealed a complex transmission landscape where relapses predominantly contribute to recurrent parasitemia. Despite a low average MOI of 1.4, this study revealed a high unique haplotype (H = 67), high average haplotype diversity (Hd = 0.799), high nucleotide diversity ((ℼ = 0.044), and 55.2% minority haplotype variants. Moreover, the overall infection Nei’s unbiased expected heterozygosity (HE) was 0.826; in paired primary and recurrent episodes, HE was 0.829 and 0.766, respectively. However, the overall polyclonal infection was 34.6%. In paired primary infection and recurrent episodes, the polyclonal infection rate was 39.0% vs 29.7%, respectively, with no statistical difference. The polyclonal infections also showed no significant difference between symptomatic or asymptomatic infection, malaria treatment group, sex, season of *P. vivax* infection, and age group. Besides these, the majority, 81.9% of recurrent paired samples showed homology, while 56.7% of paired homologous samples share identical alleles from the primary infection. On the contrary, the binomial probability showed that only 34.2% of the recurrent pairs were relapses. Minority variant expansion was observed in 20.7% of the heterologous pairs. In paired primary and relapse episodes, H01 haplotype showed a significant increase of 26.8% vs 42.6%, while H02 haplotype showed a significant decrease of 30.1% vs 21.5%, respectively. The analysis of molecular variance (AMOVA) of *P. vivax* in the overall sample was similar within individuals and among individuals (50.2% vs 49.8%). However, in paired primary and recurrent episodes, within-individual and among-individual genetic variation were (58% vs 42%) in primary and (40% vs 60%) in recurrent episodes, respectively.

Even though the study showed a low average multiplicity of infection (MOI = 1.4), the *P. vivax* parasite population exhibits relatively high genetic diversity, as evidenced by 67 unique haplotypes (H = 67), high haplotype (Hd = 0.799) and nucleotide (π = 0.044) diversity, and a high expected heterozygosity (HE = 0.826). The findings of high genetic diversity parameters were consistent with moderate-to-high malaria transmission areas studies such as Tak, Thailand ^39^, southern Mexico ^35^, Ethiopia ^27^, Papua New Guinea ^34^, and northern Cambodia ^19^. However, incongruent with the pre-elimination areas of Yala, Thailand ^39^ and Sabah, Malaysia ^26^. The higher the population level genetic diversity and low individual level diversity (MOI) may be due to this study conducted in irrigation scheme. This irrigation project attracts migrant workers to the area, and the migrant laborers potentially introduce novel parasite strains, enriching the local gene pool ^40,41^. This is further corroborated by a high proportion of minority variant haplotypes (55.2%), indicating a large population reservoir of circulating *P. vivax* parasites in the area. Since the area’s showed low malaria transmission (2.0% prevalence) ^42^, this hidden diversity poses a significant risk of outbreaks, demonstrate the need for strengthened malaria control and elimination efforts.

In paired analysis of within-host diversity of primary and recurrent episodes, the average MOI was 1.5 vs 1.4, respectively. This MOI was higher than that observed in the very low transmission settings of China ^43^, India ^24^, and Nepal ^21^. While relatively similar to several studies in low-to-moderate transmission settings across Southeast Asian and African studies, such as Sabah, Malaysia ^26^, Eastern Indonesia ^20^, Bangkok, Thailand ^24^, southern Thailand ^39^, Myanmar study ^24^, southern Ethiopia ^44^, and Jimma Zone, Ethiopia ^27^. However, it was substantially lower than the moderate-to-high transmission settings reported in Jimma, Ethiopia ^27^, Papua New Guinea ^34^, and northern Cambodia ^19^. The discrepancy between these studies might be due to differences in transmission intensity. Several lines of evidence suggest MOI is a good indicator of transmission intensity ^20,23,34^. The other possible reason could be a lack of standardization in genotyping methods ^24,34,44^. In addition, in the case of next-generation sequencing methods, the choice of sequencing platform (e.g., Illumina, 454/Roche) ^19,27^ and critical parameters like amplicon length can significantly influence MOI estimates, as evidenced by a study showing different results from short versus long amplicons sequenced from the same sample ^27^.

Furthermore, the paired analysis revealed high genetic homology (81.3%) between recurrent and preceding episode, with 55.6% of the homologous pairs sharing identical alleles. However, in the binomial probability model estimates only 34.2% were true relapses. A handful of studies have shown that analysis of primary-relapse pairs showed homologous ^18,21,26,27^. While several studies documented relapse were heterologous to the primary infection ^19,24,25,28^. The finding of homologous pairs points to the persistence of speci c parasite lineages and strongly supports hypnozoite reactivation as the predominant cause of recurrence. The observation of minority variant expansion in 20.7% of heterologous pairs also provides direct evidence for hypnozoite reactivation (relapse). The low percentage of the binomial probability model estimation of relapse might be due to a few alleles, such as H02 (30.1%) in the primary infection and H01 (42.6%) in recurrent episodes were predominant alleles and affected the randomness of the observation. Even if the sample size was very small, the reactivation of hypnozite was strengthened by the age-separated analysis, relapse episodes in children under the age of five showed 12 out of 12 (100%) homologies to the consecutive recurrent infection when the relapse episode that occurred between 29 to 60 days after the primary infection. This result is in line with a study conducted by Imwong *et al*., which documented that the first *P. vivax* relapses of life are usually genetically homologous ^28^. This finding suggests that in young and immunologically naïve children, the first *P. vivax* infection causes homologous relapses, before the cumulative effect of repeated exposures to diverse clones.

This study found an overall polyclonal infection rate of 34.6%. In paired samples, the rates for primary and recurrent episodes were similar (39.0% and 29.7%, respectively), with no statistically significant difference. This study result was higher than the Australian ^25^ and Sabah Malaysia studies ^26^ but comparable with a multicenter study of Southern Ethiopia ^44^, Papua New Guinea ^32^, and a recent study in Eastern Indonesia ^20^. However, high-rate polyclonal infections were documented in the Western Brazilian Amazon ^45^, Jimma zone, Ethiopia ^27^, Papua New Guinea ^34^, and northern Cambodia ^19^. In *P. vivax* infection, polyclonal infection could occur as a result of three distinct phenomena in such as co-transmission, superinfections, and reactivation of hypnozoites from past exposure. Co-transmission occurs when an individual is bitten by a single mosquito that carries a polyclonal parasite genotype. While superinfections occur when an individual is bitten by two or more mosquitoes that carry a unique parasite genotype, and these unique parasite clones recombine in the mosquito to form multiclonal sporozoite, then multiclonal hypnozite and parasite in the bloodstream ^23^. These two phenomena (co-transmission or superinfections) are more common in high-transmission settings. Individuals in endemic areas accumulate a diverse reservoir of hypnozoites in the liver from past exposures ^28,46^. The simultaneous reactivation of multiple latent hypnozoites from previous infections can also produce a polyclonal blood-stage infection ^19^. Hypnozoite reactivation is a key mechanism that can cause polyclonal infections even in lower transmission settings.

The analysis of molecular variance (AMOVA) further elucidates the population structure. The near-equal genetic variation within (50.2%) and among (49.8%) individuals in the overall population was seen. The dynamic shift was recorded in the paired samples of primary and recurrent episodes. More than half (58%) of the variation occurred within individuals during primary infection, aligning with the moderate polyclonal infections (39.0%) at this stage. In contrast, 40% of the variation occurred within individuals in recurrent episodes. This suggests a close related genetic relationship was found among *P. vivax* clones within individuals, and it might be due to the reactivation of hypnozoite in the liver. High homologous pairs in the primary infection and consecutive recurrent episodes in this study also corroborate this. The lower within-individual variation has been reported elsewhere ^27^.

In the present study, MOI was not significantly associated with malaria symptoms, season of *P. vivax* attack, malaria treatment group, and the sociodemographic factors such as sex and age. Studies documented varied results; some studies showed MOI increases with age and malaria symptoms ^32,34,47–49^. However, others showed no association ^27,44^. Some also reported that seasonality did not affect MOI ^34^. Malaria treatment type did not affect MOI in this study. However, the CQ-treated group showed fluctuating change in MOI values, while the CQ + PQ group demonstrated an overall decrease in MOI values. This can be explained by the fact that PQ is a hypnozoitocidal drug that reduced some of the hypnozoite clones in the liver. However, the reappearance of dominant alleles (H01 and H02), coupled with the occurrence of recurrent infection beyond day 29 after the primary attack and minority variant expansion, provides strong evidence that these cases are attributable to relapses from hypnozoites that survived primaquine therapy. This study cannot rule out the emerging drug resistance and other factors, such as inadequate dosing or poor absorption due to the PQ administration being unsupervised.

This study is not without limitations; the main one was it was used a single genetic marker to distinguish between relapse and reinfection. Although pvmsp1 is highly diverse and useful for initial genotyping, analyzing only one locus may not give true picture to distinguish between parasite populations. This could lead to underestimating the real burden of reinfection if genetically similar but distinct strains are mistaken for relapses. In addition, the study did not examine molecular markers for drug resistance. As a result, it cannot address the role of emerging drug resistance or other factors like inadequate dosing or poor absorption. The study protocol was not considered infection below day 28 to determine recrudescence due to the aim of the study was to see relapse of P. vivax. Future research that includes multi-locus genotyping and resistance marker surveillance will offer a more complete understanding of the patterns in recurrent P. vivax infections.

In conclusion, this study highlight high genetic diversity at the population level, while a low individual level diversity (MOI). Moreover, the vast majority of recurrent infections were relapses (reactivation of hypnozoites), as the infections shared genetically identical alleles (homologous), occurred beyond day 29 after the primary attack, and these findings were strengthened by the occurrence of homologous relapses in under 5 children. The study also revealed that there were predominant haplotype activation or persistence of a few haplotypes even if PQ was administered. The high population diversity and low individual level diversity differences were could be driven by the influx of migrant workers, who might have introduced a wide array of parasite genotypes, enriching the local parasite gene pool even within a setting of overall low malaria transmission. A number of unique haplotypes, high nucleotide diversity and a substantial reservoir of minority variants evidenced this. Briefly, these findings revealed that the area harbored a diverse parasite population. This cause a hidden reservoir of *P. vivax*, maintained by human migration and the parasite’s unique biological ability to relapse. This created a sustained potential for outbreaks, even when routine prevalence metrics appeared low. Therefore, there is a critical need to strengthen malaria surveillance and control strategies that specifically target mobile populations as well as the local community, and improved health education on treatment adherence. In addition, optimized the PQ regimens to reduce relapse and sustain malaria control toward elimination particularly in low transmission settings.

## Methods

### Ethics declarations

The Ethiopia National Research Ethics Review Committee (NRERC) granted ethical clearance under reference number 3.10/131/2018. The local health authorities also provided study permission, namely the Dabo Hanna District Health office (Ref. No. WF/662/19) and the Jimma Arjo District Health office (Ref. No. 0178/JA/2019). Each participant provided written informed consent or assent after receiving a detailed explanation of the study’s objectives and follow-up procedures, as well as their right to withdraw from participation at any time without penalty. Parents or legal guardians signed the informed consent form for youngsters under the age of sixteen, while persons between the ages of twelve and sixteen were additionally requested to give their own assent. All methods were performed in accordance with relevant guidelines and regulations.

### Study area and sample collection

This study was conducted in seven health facilities of the Arjo-Didessa irrigated sugarcane plantation project area and its surrounding areas of Jimma-Arjo and Dabo-Hanna districts of Oromia Region, located in southwest Ethiopia (8°36′0’’ N, 36°24′0’’ E). The seven health facilities were Arjo-Didessa Sugar factory clinic, Command 2 Health Post and Command 5 Health Post were the health facilities which gave health service for irrigation workers, while Abote-Didessa Health Post, Hunde Gudina Health Post, Kerka Health Post, and Sefera Tabiya Health Post were the health facilities for the surrounding local communities. A detailed description of the study site has been published elsewhere ^42,50^. The area has low malaria transmission and shows seasonal patterns ^42^. A total of 215 *P. vivax* cases were subsequently followed for a median of 105 days of which *P. vivax* reoccurred in 95 individuals (44.2%) from which 444 *P. vivax* positive samples were collected, including the primary attack published elsewhere ^50^. For this study, 370 *P. vivax* samples were selected from 444 *P. vivax* positive samples. Theses includes 133 patient samples with primary infection without recurrence and 82 patient samples with primary infection and one to five recurrent episode which sum-up 237 samples were amplicon deep sequenced to determine relapse of *P. vivax* malaria. The study participants ranged in age from 1 to 58, and data collection was conducted from September 2019 to July 2022. Study participants were followed at day 28, day 42, and then monthly thereafter for 12 months. During the follow-up visit, if *P. vivax* reoccurred, it was treated with chloroquine (CQ) or chloroquine plus primaquine (CQ + PQ), according to the national malaria treatment guideline for non-elimination and elimination targeted districts, respectively. In Ethiopia, malaria elimination program was launched since 2018 in 239 districts in five regions, including Oromia. In malaria elimination-targeted districts, patients received CQ plus low-dose (0.25 mg/kg daily) PQ ^51^. The non-elimination targeted districts received CQ alone. Four health facilities from the Jimma-Arjo district that provided CQ + PQ and three health facilities from the Dabo-Hanna district that provided CQ alone were included in this study. The general characteristics of 370 samples are shown in Table 4.

#### PCR amplification and deep sequencing of pvmsp1

The saponin/Chelex DNA extraction method was used according to the published protocol from dried blood spot on filter paper ^52^, by eluting the genomic DNA in a total volume of 200μl TE buffer. *Plasmodium vivax* identi cation was done by nested PCR ampli cation with species-specific primers based on the small subunit ribosomal RNA (18S rRNA) genes ^53,54^. The PCR products were prepared using a two-step PCR approach targeting the highly variable region of the *pvmsp1* gene by Illumina MiSeq sequencing [7,8]. In the first PCR reaction the gene-specific primers (forward and reverse) attached to the 5’ ends, while in the second PCR reaction the universal primers with barcode primers attached to the 5′ ends. The amplicon product was 309 base pairs (bp) with the KP759875 reference sequence.

PCR ampliflication of each sample was conducted in a 20 μl reaction mixture containing 2 μl of genomic DNA, 4 μl of 5 × PCR buffer, 1 unit of high-fidelity PrimeSTAR^®^ GXL DNA Polymerase (Takara Bio USA, Inc., Mountain View, CA), and 10 pmol of each primer. The laboratory strain *P. vivax* Pakchong (MRA-342G) was included as a control. Amplification reactions were performed with an initial denaturation at 94°C for 3 min, followed by 35 cycles at 94°C for 30 s, 55°C for 30 s, and 72°C for 60 s, with a final 6-min extension at 72°C according published protocol ^27^. Ten samples were amplified in duplicate, each with a unique barcode, to confirm the amplicon. Amplicons were cleaned and normalized to 1 ng/μl concentration using the SequalPrep Normalization Plate Kit (Thermo Fisher Scientific, Inc., Waltham, MA, USA). Amplicon deep sequencing was performed on an Illumina MiSeq platform in paired-end mode using a MiSeq Reagent Kit v3 PE300 (UCI Genomics High-Throughput Facility, Irvine, CA) with PhiX control (Illumina, PhiX Control v3) and the minimum target read depths of 10,000× according to a previously published protocol ^43^.

### Haplotype determination

Haplotypes of *pvmsp1* variants were determined by AmpSeqR, R package (https://github.com/bahlolab/AmpSeqR). The pipeline integrates several R packages and newly developed functions to filter out sequencing noise and improve the accuracy of the detected sequence data. The pipeline offers various analysis steps, including data preprocessing, amplicon sequence variant (ASV) estimation, post-processing, and data visualization, and automatically generates a comprehensive report in R Markdown that contains all essential results. This pipeline is designed to simplify bioinformatics processing, leading to a comprehensive pipeline that starts from raw FASTQ files and generates a final reproducible report ^55^. EstimateS v 9.1.0 program ^56^ was used to infer estimates of allelic richness. Sample-based rarefaction (haplotype accumulation) curves were plotted with 95% confidence intervals. The input matrix used *msp1* haplotype abundance or incidence data for a set of related samples. Relapse or reinfection of *P. vivax* was classified based on a previously published method ^19,27,57^. Homologous pairs were defined as having the same dominant or codominant haplotype at recurrence as seen in the preceding episode ^19^. Minority variant was defined parasite population existing at < 20% in-host frequency. While minority variant expansion was defined as an allele existing at < 20% in-host frequency in the primary infection reappeared as the dominant or increased > 20 in-host frequency at recurrence^19,58^. MOI, also termed as complexity of infection (COI), was defined as the number of unique *pvmsp1* haplotypes detected in a single relapse sample ^20,29,32^.

### Sequence variation analysis and haplotype relationship within multiple infection

MAFFT v7.526 online version (https://mafft.cbrc.jp/alignment/software/) was used to align DNA sequences ^59^. Sequence identity was calculated using a Bioedit v7.7.1 ^60^. DnaSP v6.12.03 was used for the analysis of haplotype and nucleotide diversity ^61^. The Nei’s unbiased expected heterozygosity (HE) was calculated as a measure of overall genetic diversity for each genotype method ^62^. Analysis of Molecular Variance (AMOVA) was conducted by GenAlEx 6.51b2 to estimate sequence variation within and between infections ^63^. The MEGA v11 was used to create a UPGMA phylogenetic tree ^64^. The PopART v1.7 software was used to construct a median spanning haplotype network between haplotypes ^65^. Median comparisons for MOI were computed using the Mann-Whitney test from primary infection vs recurrent episodes, asymptomatic vs symptomatic infection, and between females and males, and between CQ-treated and CQ + PQ, while the Kruskal-Wallis test was used for age group by Graph Pad Prism v9.5.1.

## Supplementary Material

This is a list of supplementary files associated with this preprint. Click to download.

• SupplementaryHG.docx

• PopulationfrequencysamplecountsandGenBankblastresultsofthe67haplotypesidentified.xlsx

• Amplicondeepsequencingdataof370P.vivaxsamples.xlsx

• Supplementaryinformation.docx

## Figures and Tables

**Table 1:** Age and time separated analysis of homologous and heterologous pairs

Age group (years)	< 5		5–15		> 15	
Time of recurrence (days)	29–60 n (%)	> 60 n (%)	29–60 n (%)	> 60 n (%)	29–60 n (%)	> 60 n (%)
**Homologous pairs**	12 (100)	15 (71.4)	9(81.8)	35(87.5)	14(82.4)	11 (20.4)
**Heterologous pairs**	0	6 (28.6)	2(18.2)	5(12.5)	3 (17.6)	43 (79.6)
**Total,**	12 (100)	21 (100)	11 (100)	40 (100)	17 (100)	54 (100)
**p-value**	0.041*		0.628		0.806	

Significant at * *p < 0.05*

**Table 2:** Results of MOI of *pvmsp1* by amplicon deep sequencing from the overall and paired isolates

Parameters	Overall isolates	Paired isolates	
		Primary	Recurrence
Number of subjects	215	82	82
Number of samples	370	82	155
Median MOI [IQR]	1[1–2]	1[1–2]	1[1–2]
Mean MOI	1.42	1.5	1.35
Max MOI	4	3	3
No. polyclonal	128	32	46
% Polyclonal	34.6	39.0	29.7
No. haplotypes	67	28	39
No. private haplotypes		15	26
H01 haplotype: n (%)	151 (40.8%)	29 (26.8%)	81 (42.6%)
H02 haplotype: n (%)	127 (34.3%)	36 (30.1%)	43 (21.5%)
Heterozygosity (HE)	0.826	0.829	0.766

**Table 3:** Analysis of molecular variance (AMOVA) of *P. vivax* infections using *pvmsp1* deep sequencing in overall, paired primary infection, and recurrent episodes

Infection	Source of variation	df	SS	MS	Est. variance	Variation (%)	*p*-value
**Overall**	Among individuals	127	1640.18	12.91	4.02	49.8%	0.001
	Within individuals	154	626.17	4.06	4.06	50.2%	
	Total	281	2266.35		8.08	100%	
**Primary infection**	Among individuals	77	954.01	12.39	3.44	42%	0.001
	Within individuals	95	451.50	4.75	4.75	58%	
	Total	172	1405.51		8.20	100%	
**Recurrent episode**	Among individuals	49	618.53	12.62	4.43	60%	0.001
	Within individuals	59	174.67	2.96	2.96	40%	
	Total	108	793.19		7.39	100%	

**Table 4:** Characteristics 370 samples

Variable	Number (n)	Percent (%)
**Age**		
< 5	54	14.6
5–15	108	29.2
> 15	208	56.2
**Sex**		
Female	112	30.3
Male	258	69.7
**Treatment**		
CQ only	174	47
CQ + PQ	196	53
**Patient status**		
Asymptomatic	117	31.6
Symptomatic	253	68.4
**Season of sample collection**		
Dry	138	37.3
Wet	232	62.7
**Infection status**		
Primary infection (at enrollment)	215	58.1
Recurrent infections (follow-up)	155	41.9

## Data Availability

The datasets generated and/or analysed during the current study are available in the NCBI repository with a Web link https://www.ncbi.nlm.nih.gov/nuccore/PX841045 https://www.ncbi.nlm.nih.gov/nuccore/PX841046 https://www.ncbi.nlm.nih.gov/nuccore/PX841047 https://www.ncbi.nlm.nih.gov/nuccore/PX841048 https://www.ncbi.nlm.nih.gov/nuccore/PX841049 https://www.ncbi.nlm.nih.gov/nuccore/PX841050 https://www.ncbi.nlm.nih.gov/nuccore/PX841051 https://www.ncbi.nlm.nih.gov/nuccore/PX841052 https://www.ncbi.nlm.nih.gov/nuccore/PX841053 https://www.ncbi.nlm.nih.gov/nuccore/PX841054 https://www.ncbi.nlm.nih.gov/nuccore/PX841055 https://www.ncbi.nlm.nih.gov/nuccore/PX841056 https://www.ncbi.nlm.nih.gov/nuccore/PX841057 https://www.ncbi.nlm.nih.gov/nuccore/PX841058 https://www.ncbi.nlm.nih.gov/nuccore/PX841059 https://www.ncbi.nlm.nih.gov/nuccore/PX841060 https://www.ncbi.nlm.nih.gov/nuccore/PX841061 https://www.ncbi.nlm.nih.gov/nuccore/PX841062 https://www.ncbi.nlm.nih.gov/nuccore/PX841063 https://www.ncbi.nlm.nih.gov/nuccore/PX841064 https://www.ncbi.nlm.nih.gov/nuccore/PX841065 https://www.ncbi.nlm.nih.gov/nuccore/PX841066 https://www.ncbi.nlm.nih.gov/nuccore/PX841067 https://www.ncbi.nlm.nih.gov/nuccore/PX841068 https://www.ncbi.nlm.nih.gov/nuccore/PX841069 https://www.ncbi.nlm.nih.gov/nuccore/PX841070
